# Orthogeriatric co-management and incident nursing home admissions in older patients with fragility fractures other than the hip—a retrospective cohort study using insurance claims data from Germany

**DOI:** 10.1186/s12916-025-04073-3

**Published:** 2025-04-29

**Authors:** Daniel Schoene, Kilian Rapp, Patrick Roigk, Clemens Becker, Andrea Jaensch, Claudia Konnopka, Hans-Helmut König, Thomas Friess, Gisela Büchele, Dietrich Rothenbacher

**Affiliations:** 1 Department of Clinical Gerontology, Robert Bosch Hospital, Auerbachstr. 110, Stuttgart, 70376 Germany; 2 Institute of Epidemiology and Medical Biometry, Ulm University, Ulm, Germany; 3 Unit of Digital Geriatric Medicine, Heidelberg University Hospital, Heidelberg, Germany; 4 Department of Health Economics and Health Services Research, University Medical Center Hamburg-Eppendorf, Hamburg, Germany; 5 AUC - Akademie Der Unfallchirurgie GmbH, München, Germany; 6 Center for Trauma Research, Ulm University, Ulm, Germany

**Keywords:** Aged, Geriatrics, Fractures, Acute care, Hospital, Orthogeriatric co-management, Homes for the aged, Nursing home admission, Claims data, Retrospective cohort

## Abstract

**Background:**

Orthogeriatric co-management (OGCM) has been proposed as care model for geriatric patients with fragility fractures. However, its impact on nursing home (NH) admissions following non-hip fractures is unclear. This study aims to assess the association between OGCM and the probability of NH admissions within 6 months in older patients with fragility fractures other than the hip.

**Methods:**

This retrospective cohort study utilized nationwide insurance claims data from Germany (from years 2014–2018), covering individuals aged 80 years or older with fractures of the humerus, forearm, pelvis, or vertebrae. Based on the number of OGCM claims per year, hospitals were categorized as either OGCM or no OGCM. The primary outcome was the incidence of NH admissions within 6 months of the index fracture. Quasi-Poisson regression models were used to calculate incidence rate ratios (IRRs) with 95% confidence intervals (CI), adjusted for age, sex, prior care needs, comorbidity score, and rehabilitation transfer rates.

**Results:**

A total of 106,217 patients were included in the analysis. The incidence of NH admissions varied by fracture site, ranging from 11.1% for pelvic to 24.7% for vertebrae fractures, respectively. OGCM was associated with a reduced probability of NH admissions for humerus fractures (IRR 0.94, 95% CI 0.88–1.00) and vertebral fractures (IRR 0.92, 95% CI 0.87–0.97). No statistically significant associations were found for forearm (IRR 1.06, 95% CI 0.97–1.15) or pelvic fractures (IRR 1.02, 95% CI 0.96–1.09).

**Conclusions:**

OGCM went along with a reduced probability of NH admissions in geriatric patients with humerus and vertebral fractures but showed no statistically significant benefit for forearm or pelvic fractures. The results highlight the need for targeted OGCM strategies based on fracture type and patient demographics to optimize outcomes in this vulnerable population.

**Graphical Abstract:**

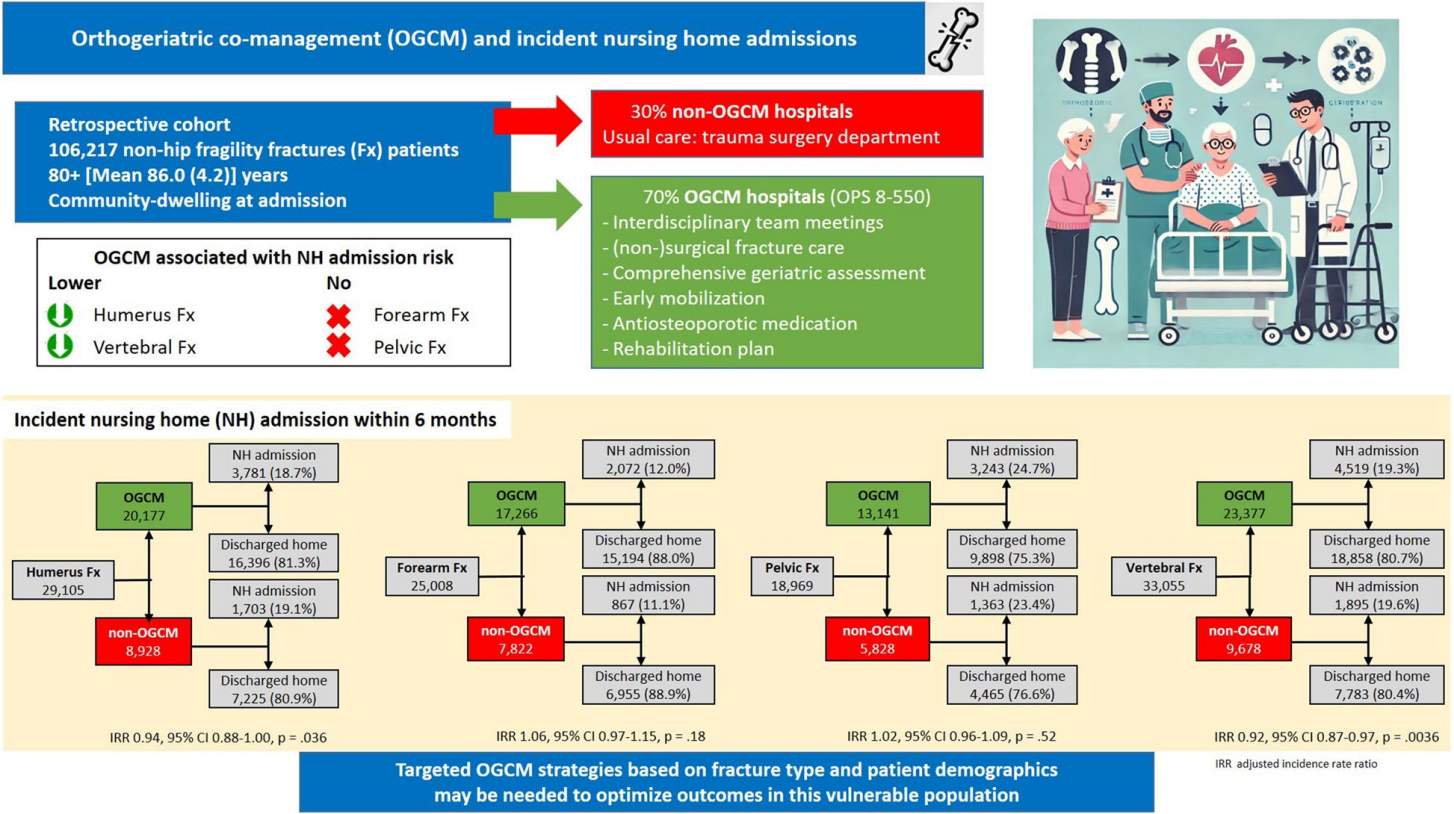

**Supplementary Information:**

The online version contains supplementary material available at 10.1186/s12916-025-04073-3.

## Background

Age-related fragility fractures are a major challenge in the context of an aging global population. Apart from increased mortality, fragility fractures may lead to numerous negative health consequences including pain, mobility disability, reduced quality of life, and care dependency [1–7]. These fragility fractures mostly result from low-trauma falls in combination with a decline in bone strength and the age-associated increase in osteoporosis prevalence and severity. About one-third of community-dwelling adults aged 65 years or older fall at least once a year, a number that reaches 50% around the age of 80 [8, 9]. As individuals age, the incidence of fall-related fractures disproportionately increases [10], which can only be partially explained by the reduction in bone strength. With advancing age, physiologically driven changes increase the likelihood of biomechanically unfavorable falls that exert a high impact on the bone, contributing to a greater risk of fractures [11]. Older fracture patients hence typically present complex clinical profiles characterized by multiple geriatric syndromes, including frailty, multimorbidity, sarcopenia, and cognitive impairment, underscoring the necessity for multifaceted intervention strategies.

Orthogeriatric co-management (OGCM) has emerged as a leading care model in addressing this challenge. OGCM is a collaborative approach that involves the joint management of older fracture patients by both, geriatricians and orthopedic surgeons within a multi-disciplinary team [12]. This model aims to provide comprehensive care that addresses the multifaceted needs of this patient group [12]. OGCM encompasses a comprehensive geriatric assessment that prioritizes early mobilization and includes the administration of antiosteoporotic medication as required. The treatment aims to provide tailored care to older trauma patients, focusing on restoring function, minimizing disability, and enhancing overall health to prevent future injuries. Inpatient rehabilitation, which begins shortly after hospital admission, involves regular team meetings to ensure comprehensive patient management and is meticulously planned with clearly defined functional goals, particularly addressing geriatric syndromes. The goal is to implement an early intervention strategy that significantly impacts the patients’ recovery and independence in activities of daily living (ADL). Existing data on OGCM, predominantly focusing on hip fracture patients, have demonstrated its benefits in reducing mortality rates, length of hospital stay, and delirium risk [13, 14]. The findings underscore the potential benefits of integrated care models in treating complex, age-related conditions.

Maintaining functional levels that ensure independent living is of utmost importance for older adults [15–18]. With regard to functional outcomes, to date, very low to moderate certainty of evidence suggests that OGCM may improve ADL, walking ability, and physical capacity measures, such as the Short Physical Performance Battery [13, 14, 19, 20]. However, only few studies conducted in hip fracture patients have been published on the probability of losing independence, and hence, the need of institutionalized care thereafter. A small randomized controlled trial conducted in Taiwan with 162 hip fracture patients found no difference in the number of people admitted to nursing homes (NH) between the group treated by a multidisciplinary team and the control group at 12 months [21]. In a single-center study with historic controls, there was also no difference in the 12-month residential status detected between the group receiving OGCM and the no OGCM group [22]. Finally, in a previous claims data analysis, we were unable to demonstrate an association of OGCM on NH admissions within 6 months of hospital admission in more than 23,000 hip fracture patients aged 80 years or older, although short-term mortality was decreased significantly [23]. Given the high incidence of fragility fractures at various anatomical sites in older patients, their severe consequences, and the presence of site-specific risk factors, greater attention should be directed toward other osteoporotic fracture sites [2, 24–27]. The institutionalization rates following spine and pelvic fractures are not different compared to hip fractures and thus appear to be associated with functional decline [1]. However, to date, there are no data available that compare the probability of admission to NH in older patients with fragility fractures other than the hip, treated in hospitals with or without OGCM.

Therefore, the objective of this study was to investigate the probability of incident NH admissions within 6 months in geriatric patients with a fracture of the humerus, forearm, pelvis, or vertebra treated in hospitals with or without OGCM using national insurance claims data.

## Methods

### Study design

For this retrospective cohort study, nationwide insurance claims data were provided by the research institute of the largest health insurance in Germany (Wissenschaftliches Institut der AOK (WIdO)) covering about one-third of the population for the years 2014–2018.

### Study population

Included patients fulfilled the following criteria:

1. Health insurance with the company “Allgemeine Ortskrankenkasse” (AOK).
2. Aged 80 years or older.
3. Admitted to hospital and diagnosed (ICD-10 coded) with non-hip osteoporotic fracture during the index period 2014–2018. Where the index hospital stay commenced in 2018 and extended into 2019, it was included in the analysis (data available up to June 30, 2019).
  The following fractures were included and considered osteoporotic (ICD-10 codes in brackets): humerus (S42), forearm (S52), pelvis (S32.1, S32.3, S32.4, S32.5, S32.81, S32.83), vertebral bodies (S12.0, S12.1, S12.2, S12.7, S12.9, S22.0, S22.1, S32.0). Osteoporotic fractures were identified based on the main discharge diagnosis. In addition, we considered osteoporosis with pathological fracture (M80) as the primary discharge diagnosis, plus one of the aforementioned fractures in the main admission or additional diagnosis. For each type of fracture, only the first fracture per person was considered. The start of the index stay was considered the date of hospital admission with the corresponding diagnosis. The index hospital was always the first hospital with the fracture diagnosis. The sample was stratified by fracture sites.
4. Available data for the outcome “NH admission within 6 months after the index fracture.”

Patients admitted to the hospital from NH were excluded, as the study aimed to investigate the incidence of NH placements following fractures. Also excluded were patients who did not survive the hospital or subacute rehabilitation stay. Furthermore, hospitals were excluded (*n* = 550) if they transferred more than 5% of their total fracture patients to avoid misclassification of exposure and selection bias.

### Study exposure

All patients underwent usual care. OGCM was defined at the hospital level rather than at the individual patient level. In Germany, fragility fractures, including those managed conservatively, are primarily treated in trauma surgery departments. OGCM is implemented within these settings to ensure interdisciplinary management by trauma surgeons and geriatricians, supported by a specialized multidisciplinary team comprising physiotherapists, occupational therapists, trained nurses, and social workers. This co-management is delivered through either a dedicated orthogeriatric unit (shared responsibility model) or a geriatric liaison service within the trauma surgery department, often with early transfer to a geriatric unit when needed.

In this study, OPS 8–550 was used as the available claims-based proxy for OGCM. OPS 8–550 represents early complex geriatric rehabilitation with structured interdisciplinary management. The procedure requires a minimum treatment duration of 7, 14, or 21 days, a standardized geriatric assessment, regular interdisciplinary team meetings, early mobilization, and a rehabilitation plan targeting functional recovery. While OPS 8–550 is formally categorized as a rehabilitation code, its use in trauma surgery units reflects structured interdisciplinary co-management rather than rehabilitation alone.

Hospitals that claimed at least 10 cases of OPS 8–550 annually were classified as OGCM hospitals, following established methodology in claims-based research and as consented by the data providers. This hospital-level classification was chosen to reduce misclassification bias, which could occur if OGCM status were determined at the individual patient level, given that OPS 8–550 can only be claimed when a predefined treatment duration is completed. Although not all patients in OGCM hospitals necessarily received OPS 8–550, they were treated in hospitals that systematically integrate geriatric expertise into trauma care pathways and promote structured co-management.

OGCM was implemented regardless of whether surgery was performed. Even in non-surgical cases, trauma surgeons remained responsible for fracture management and collaborated with geriatricians to determine optimal treatment pathways, pain management strategies, and rehabilitation needs.

While hospitals that meet OGCM criteria systematically claim OPS 8–550, some hospitals may provide limited elements of co-management without billing it. However, this is unlikely to be systematic, as OPS 8–550 is the standard mechanism for documenting interdisciplinary orthogeriatric rehabilitation in claims data and provides additional reimbursement through the German Diagnosis-Related Groups (G-DRG) system. Thus, even in the absence of direct OPS 8–550 coding for some patients, those in OGCM hospitals were expected to benefit from the structured interdisciplinary model embedded in these hospitals.

### Outcome measures

The outcome of this study was an incident NH admission within 6 months following an index fracture. The residential status was assessed monthly. The evaluation of a new NH admission started at the first month after the fracture. Patients were followed for 6 months or until death (censoring).

### Covariates

Additional data were used as covariates: (1) age; (2) sex; (3) care need in the month prior to index fracture, based on classification of the care needs within the German long-term care insurance system; (4) medication-based co-morbidity utilizing pharmacy claims data to assess patients’ chronic disease status by analyzing the number of medications prescribed in the 12 months prior to the index fracture [28]; (5) admission rate to subacute rehabilitation within 4 weeks after discharge from the index hospital; and (6) size of the index hospital.

### Statistical analysis

Uni- and multivariable quasi-Poisson regression models stratified by fracture site were utilized to analyze the data. Incidence rates were calculated. For differences between groups, incidence rate ratios (IRR) and their respective 95% confidence intervals (95% CI) were calculated. Multivariable models were adjusted for age at index fracture (continuous, standardized), sex, care need in the month prior to fracture (yes/no), percentage of subacute rehabilitation (as a categorical variable: zero percent separately, then divided into quartiles), size of the index hospital (continuous, standardized), and medication-based comorbidity score (continuous, standardized).

In addition, pre-planned subgroup analysis by age groups (80–84, 85–89, 90 + years) and sex was conducted. Sensitivity analyses were performed by only including those patients who underwent surgical intervention. Statistical analyses were performed using SAS version 9.4 (SAS Institute Inc, Cary, NC) and R version 4.3.2 (R Foundation for Statistical Computing, Vienna, Austria).

## Results

**Fig. 1:**
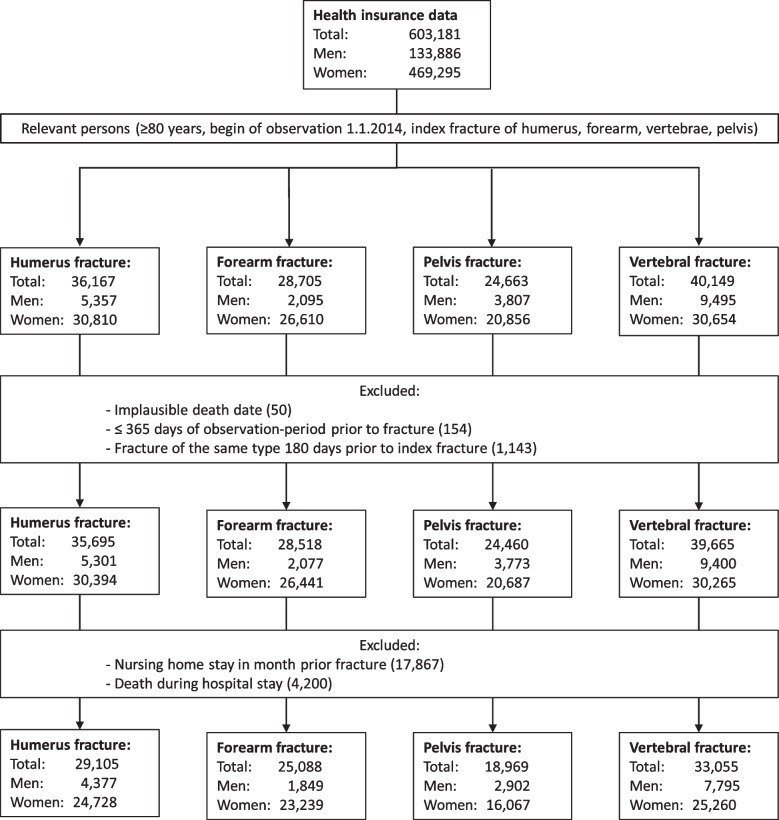
Flow chart of the participants’ selection process

We identified 603,181 patients from the insurance claims database. After applying the eligibility criteria, a total of 106,217 patients were included in the analyses. Of these, 29,105 were hospitalized for a humerus fracture, 25,088 for a forearm fracture, 18,969 for a pelvic fracture, and 33,055 had vertebral fractures. A detailed study flow is depicted in Fig. 1.

The overall mean age of participants was 86.0 years (standard deviation [SD] = 4.2 years) with only slight differences between fracture sites. Further patient characteristics are presented in Table 1. On average, participants had four comorbidities (SD = 2). The distribution of patients in the OGCM and no OGCM groups was consistent across fracture sites, with about 70% of patients treated in OGCM hospitals and 30% in hospitals without OGCM. Overall, the rate of transfer to subacute rehabilitation differed between fracture sites, with only 1.6% of individuals with forearm fractures being transferred, compared to 9.4% with humerus fractures, 14.7% with vertebral fractures, and 19% with pelvic fractures (data not in table). These rates were similar between groups, with slightly more transfers occurring from no OGCM hospitals. The proportion of individuals treated in OGCM hospitals for whom the OPS 8–550 was claimed varied overall between 7.8% for forearm fractures and 29.7% for pelvic fractures, showing a noticeable increase with advancing age (Additional file 1: Table 1).

**Table 1:** Patients’ characteristics

	Humerus fracture	Forearm fracture	Pelvis fracture	Vertebral fracture
Number patients	29,105	25,088	18,969	33,055
Age (mean, SD)	85.9 (4.2)	85.5 (3.9)	86.9 (4.4)	85.9 (4.1)
Female (*n*, %)	24,728 (85.0)	23,239 (92.6)	16,067 (84.7)	25,260 (76.4)
Medication-based comorbidity score (mean, SD)	4.0 (2.0)	3.9 (2.0)	4.3 (2.0)	4.5 (2.0)
Surgical care (%)	63.9	86.6	5.7	34.2
No OGCM (*n*, %)	8928 (30.7)	7822 (31.2)	5828 (30.7)	9678 (29.3)
* N* hospitals	318	327	310	355
- Resident of NH in the month before fracture (*n*)^a^	1601	957	1343	1473
- Rate of transfers to subacute rehabilitation (%)	10.0	1.6	20.8	16.9
OGCM (*n*, %)	20,177 (69.3)	17,266 (68.8)	13,141 (69.3)	23,377 (70.7)
* N* hospitals	515	510	521	539
- Resident of NH in the month before fracture (*n*)^a^	3718	2211	3085	3479
- Rate of transfers to subacute rehabilitation (%)	8.8	1.6	17.1	12.4
- Percentage of OPS 8–550	20.0	7.8	29.7	22.1
NH admission within 6 months (%)	18.8	11.7	24.4	19.4
Time (days) from fracture to NH admission (median, IQR)	35 (21–61)	35 (18–64)	36 (21–64)	40 (22–72)

Numbers in brackets indicate percentages; ^a^Persons were excluded from the further analysis and are not included in the number of individuals in the table

*OGCM *Orthogeriatric co-management, *NH *Nursing home, *SD *Standard deviation

**Fig. 2:**
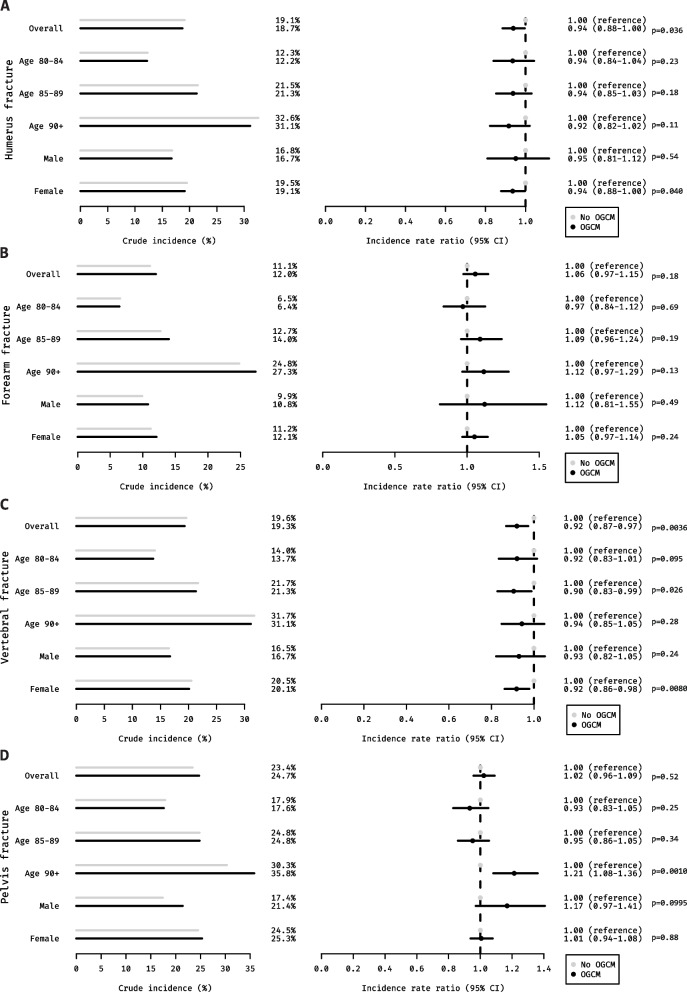
**A**–**D** Crude incidences and adjusted rate ratios for OGCM (black) versus no OGCM (gray). Data are presented as overall and stratified by age and sex according to fracture site: (**A**) humerus fracture, (**B**) forearm fracture, (**C**) vertebral fractures, (**D**) pelvic fractures. Incidence rate ratios are adjusted for age at index fracture, sex, care need in the month prior to fracture, percentage of subacute rehabilitation, size of the index hospital, and Huber comorbidity score. OGCM, orthogeriatric co-management; CI, confidence interval

The overall incidence of NH admissions after index fracture within 6 months ranged from 11.1 to 24.7% and differed more by fracture site than by treatment group (Fig. 2A–D). There was a trend for increased incidences with increasing age and for women. Among individuals with fractures, most remained in the NH until the end of follow-up or death (humerus: 82.5%, forearm: 84.5%, pelvis: 84.7%, vertebral: 88.5%). Among those who did not remain institutionalized, the duration of their stay varied, but increased from less than 2.5% for 1 month to more than 35% for 3 months across all fracture sites.

After adjustments (crude values presented in Additional file 2: Table 2), relative risks for NH admissions were reduced in patients treated in hospitals with OGCM for humerus fractures (IRR 0.94 [95% CI 0.88–1.00], *p* = 0.036) and vertebral fractures (IRR 0.92 [95% CI 0.87–0.97], *p* = 0.004). For both fracture sites, subgroup analyses by age and sex demonstrated similar point estimates across groups, however with more precise results for women and younger patients. No significant associations were observed for forearm fractures (IRR 1.06 [95% CI 0.97–1.15], *p* = 0.18) or any of the subgroups. For pelvic fractures, also no differences between groups were observed (IRR 1.02 [95% CI 0.96–1.09], *p* = 0.52). However, a marked increase in the probability of NH admission was demonstrated for the age group 90 + years (IRR 1.21 [95% CI 1.08–1.36], *p* = 0.001) and an increased point estimate for men (IRR 1.17 [95% CI 0.97–1.41], *p* = 0.10), however, the 95% CI included the null-effect value.

Sensitivity analyses partially corroborated the findings of the primary analyses (Additional file 3: Fig. S1A–D). When focusing exclusively on individuals who underwent surgical treatment, overall associations were consistent. Discrepancies in the size of group differences were noted between this sensitivity analysis and the main analysis depending on age group and sex. However, except for vertebral fractures, all comparisons showed non-significant associations. Here, in the youngest age group, individuals treated in OGCM hospitals exhibited a 16% reduced probability versus the non-OGCM group (IRR 0.84 [95% CI 0.71–1.00], *p* = 0.048), compared to an 8% reduction in the main analysis. In males, the relationship changed from 7% (IRR 0.93 [95% CI 0.82–1.05]) in the main analysis to 24% (IRR 0.76 [95% CI 0.61–0.96], *p* = 0.020) in surgically treated patients. For pelvic fractures, substantial discrepancies were observed compared to the main analysis, particularly in the middle and oldest age groups, where non-significant increases in NH admission probability of 40% and 81% were noted. However, these estimates were highly imprecise, as indicated by the wide confidence intervals (85–89 years: IRR 1.40 [95% CI 0.89–2.21], reflecting a 45% difference from the main analysis; 90 + years: IRR 1.81 [95% CI 0.75–4.36], reflecting a 60% difference from the main analysis).

## Discussion

This large retrospective cohort study, utilizing insurance claims data, aimed to determine the association between orthogeriatric co-management (OGCM) and the probability of new nursing home (NH) admissions in geriatric patients within 6 months following an index fragility fracture other than the hip. Our results demonstrate a substantial incidence of NH admissions across different types of fractures. Treatment in OGCM hospitals was associated with reduced NH admission rates for humerus and vertebral fractures, with no associations for fractures of forearm and pelvis. An increased probability of NH admission was identified for pelvic fractures in the oldest age group.

In Western nations, 10–20% of patients are institutionalized following a hip fracture, with an age-related increase in incident NH admissions [4, 29]. In our study, we observed similar NH admission rates for non-hip fragility fractures, underscoring their substantial impact on institutionalization risk. While hip fractures are often considered the most debilitating type of fragility fracture, this highlights that major osteoporotic fractures, regardless of site, carry a significant risk of institutionalization, although the care pathways and rehabilitation needs differ.

A recent meta-analysis demonstrated that multidisciplinary geriatric care in hospitals is protective against NH admissions, thus confirming the importance of the care framework central to OGCM [30]. However, OGCM was originally developed and studied primarily in hip fracture populations, where early surgery, standardized rehabilitation protocols, and discharge planning play a major role. In contrast, our findings suggest that the impact of treatment in hospitals with OGCM on NH admissions varies depending on the fracture site, likely due to differences in treatment pathways, recovery trajectories, and the degree of functional impairment associated with each fracture type. These findings emphasize the need for tailored OGCM frameworks to address the specific challenges of non-hip fragility fractures. Fractures vary in how severely they impact physical function and subsequent care needs. Notably, reductions in NH admissions of 6–10% were observed for humerus and vertebral fractures in individuals treated in hospitals with OGCM, suggesting that this multidisciplinary approach can address the complex needs of these patients. It potentially preserves or enhances their function by mitigating the risk of impairments related to (instrumental) activities of daily living, history of falls, cognitive impairments, dementia, delirium, malnutrition, and hypoalbuminemia [31]. Apart from the total sample, we identified significant group differences only in women and younger patients; however, consistent point estimates indicate similar associations across groups. Differences in significance may be explained by increased imprecision due to low power, especially in men. Vertebral and humerus fractures may limit mobility more than forearm fractures as indicated also by the larger proportion of people receiving treatment in hospitals with OGCM, hence benefiting more from the comprehensive care provided by OGCM [6].

In contrast, no differences between hospitals with OGCM compared to hospitals without on NH admission were observed for pelvic and forearm fractures. This variation may be explained by the severity and functional implications of these fracture types. Less than 2% of patients with forearm fractures received subacute rehabilitation, suggesting a less pronounced impact on daily functioning. Furthermore, a lower incidence of preoperative and postoperative complications, such as delirium, urinary tract infection, sepsis, and pneumonia, compared to hip fractures has been reported [32]. It is also known that younger, healthier, and more active individuals tend to fracture their forearms [24]. These factors likely contribute to the lower rate of NH admissions observed for forearm fractures compared to other fractures and the missing benefit of treatment in hospitals with OGCM compared to hospitals without as its complexity may not be required for this fracture type. However, the recovery trajectory for pelvic fractures, which are predominantly managed non-surgically, may differ from other types of fragility fractures investigated in the current analysis due to their profound impact on weight-bearing and mobility, aspects that are critical to maintaining independence in older adults. Functional impairments, such as decreased abilities to transfer, stand, and sit, are significant risk factors for NH admissions and are more likely caused by pelvic fractures [33]. Consequently, even with OGCM, the inherent challenges associated with the healing and rehabilitation of pelvic fractures may necessitate longer or more intensive care regimens that extend beyond the standard OGCM protocols. The probability of NH admissions for patients with pelvic fractures was significantly higher in the oldest age group (90 + years), a trend that was even higher in those undergoing surgical treatment. This latter finding should be regarded with caution as power was low with less than 6% of patients being surgically treated. Poor pre-fracture health status and comorbidities (e.g., cardiovascular, dementia, diabetes, renal dysfunction) are linked to increased risks of surgical and medical complications as well as general functional decline [6]. These frailer patients might be selected for OGCM due to their potential to benefit from comprehensive care, introducing the possibility of confounding by indication, which could diminish a potential benefit of OGCM. Alternatively, the extended time spent in OGCM might allow the multidisciplinary team to assess that an independent life at home is no longer feasible.

In the current analysis, the rates of transfer to subacute rehabilitation differed markedly across fracture sites. For instance, patients with vertebral, humerus, or pelvic fractures exhibited higher rehabilitation transfer rates compared to those with forearm fractures. This discrepancy likely reflects the more severe functional impairments associated with these fractures, which benefit more from intensive rehabilitation. However, the absence of differences in rehabilitation transfer rates between the OGCM and no OGCM groups across fracture types, combined with our statistical model that controlled for transfer rates to subacute rehabilitation, suggests that the benefit of treatment in hospitals with OGCM in reducing NH admissions may not be due to increased access to rehabilitation, but rather to the quality and integration of care processes.

Findings from hip fracture patients indicate that OGCM is associated with improved functional outcomes [13, 14, 19, 20] without a change in residential status [21–23]. While the current results show that several factors need to be regarded, it may be that moving to a NH is a positive outcome for individuals identified by the OGCM team as unable to live independently. While lower NH admission rates are often viewed as an indicator of treatment success, it is important to recognize that NH placement can sometimes be beneficial and the results of an adequate discharge management. For vulnerable, dependent individuals, NHs can provide enhanced safety and improved quality of life [34]. Thus, OGCM’s role extends beyond reducing NH admissions; it also involves identifying patients for whom home or community-based care is no longer safe or viable.

### Strength and limitations

To our knowledge, this is the first analysis to investigate the association between treatment in hospitals with OGCM and incident NH admissions in older patients with fragility fractures other than the hip compared to hospitals without OGCM. Our study’s external validity is reinforced by the use of a large, nationwide health insurance database covering about one-third of the German population. Another strength is that we have no response bias in claims data. Therefore, this extensive data set provides a robust basis for evaluating the potential benefits of treatment in hospitals with OGCM across a representative sample of very old people.

This study is subject to several limitations that must be considered when interpreting the results. First, the findings are constrained by the observational nature and the retrospective cohort design, which limits our ability to infer causality between OGCM and NH admissions. Second, although we have adjusted for various confounders, unmeasured variables, particularly at the patient level, could influence the outcomes. Third, the exclusion of patients who did not survive the hospital stay might have introduced survival bias. This could potentially lead to an overestimation of NH admissions in OGCM hospitals, as frail individuals who survive are more likely to be discharged to NHs. However, our recent analysis did not show higher survival rates in hospitals with OGCM [35]. Fourth, only fractures requiring inpatient care were considered in this study. For instance, uncomplicated forearm fractures that can be managed on an outpatient basis are not represented in our dataset. Fifth, we did not differentiate between permanent NH admission and temporary stays due to data constraints. While our findings indicate that most individuals remain institutionalized following a fracture, we cannot exclude the possibility that some patients who initially returned home may have subsequently been admitted to a NH permanently. Future studies with more granular longitudinal tracking of care transitions are needed to better capture these trajectories. Sixth, exact daily data on NH admission were not available, as only monthly records were provided. While we accounted for time from fracture to admission in our model, time-to-event analyses such as Cox regression were not feasible. This limits the ability to distinguish between early and delayed admissions and to accurately assess the temporal relationship between fracture occurrence, institutionalization, and potential competing events such as death. Finally, the classification of the hospitals into OGCM and no OGCM, based on the presence of a specific procedural code, may not fully capture the extent of geriatric care provided. This classification was implemented at the hospital level rather than for individual patients, which is pivotal as it helps mitigate the risk of misclassification. Hospitals implementing OGCM are likely to foster an environment where multidisciplinary care is emphasized, potentially benefiting all patients, not just those receiving a specific intervention. However, this method assumes a homogeneity of care quality within hospitals that might not exist. Moreover, it does not account for the possibility that some patients in OGCM hospitals might not receive OGCM. These are more likely younger, and less cognitively and functionally impaired individuals [36]. Consequently, the benefits of OGCM might have been underestimated.

### Implications

Future research should prioritize prospective studies to corroborate these findings and explore the mechanisms by which treatment in hospitals with OGCM influences outcomes across diverse patient populations. There is a critical need to optimize and tailor OGCM protocols to address the unique challenges posed by different fracture types, including the specific mechanisms by which surgical and conservative treatments may affect recovery trajectories in older fracture patients. Investigating the variability in OGCM implementation across hospitals could provide insights into optimizing this care model for broader and more effective applications. Furthermore, investigating how certification for specific Geriatric Trauma Centers (“Alterstraumazentrum [ATZ]” in German), which meet stringent criteria for orthogeriatric care, affects NH admissions after fractures could reveal ways to enhance OGCM protocols, potentially improving patient outcomes. Future studies could benefit from a more granular approach that includes both hospital- and individual patient-level data. Such analyses would allow researchers to more accurately attribute outcomes to OGCM by directly linking patient characteristics with specific interventions, thereby providing a clearer picture of the benefits of OGCM. Finally, studies should investigate whether the value of OGCM lies in identifying individuals who are incapable of living independently and who may subsequently experience a better quality of life in a NH.

## Conclusions

Our findings demonstrate that treatment in hospitals with OGCM is associated with a reduced probability of NH admissions for certain fracture types, specifically humerus and vertebral fractures, in adults aged 80 years and older. However, the benefit is not uniform across all fracture types or patient demographics, indicating a need for targeted strategies that consider the specific needs and risks associated with different fractures and patient groups.

## Supplementary Information

Additional file 1: Table 1. The proportion of individuals in the OGCM group for whom the OPS 8 - 550 was claimed separated by fracture type.

Additional file 2: Table 2. Crude incidence rate ratiosfor OGCM versus no OGCM overall and stratified according to fracture site.

Additional file 3: Fig. S1 A–D. Sensitivity analyses with including surgically treated patients only.

Additional file 4. STROBE checklist.

## Data Availability

The data that support the findings of this study are available from the German statutory health insurance AOK who owns the datasets supporting the conclusions of this article. Since public deposition of the data would breach ethical and legal compliance, data are only available upon formal request from the research institute of the AOK (WIdO). To request the data please contact the institutional body of the WIdO (wido@wido.bv.aok.de). In order to fulfill the legal requirements to obtain that kind of data, researchers must obtain permission for a specific research question from the German Federal (Social) Insurance Office. Additionally, researchers must conclude a contract with the statutory health insurance regarding data access which can be requested from the “AOK-Bundesverband GbR” (Federal Association of Local Health Insurance Funds) under http://aok- bv. de/kontakt/. The licensee is permitted to use the data for the purpose of the research proposal within their company, exclusively. Thereby, a company is defined as an economic unit. Licensees are not allowed to pass the data to a third party or to create Software or databases with the exception of scientific publications. Moreover, the study has to be approved by the data protection officer.

## Abbreviations

ADL: Activities of daily living
AOK: Allgemeine Ortskrankenkasse; largest health insurance company in Germany
CI: Confidence interval
IRR: Incidence rate ratio
NH: Nursing home
OGCM: Orthogeriatric co-management
WIdO: Wissenschaftliches Institut der AOK; research institute of the AOK
