# Supplementary material for: Orthogeriatric co-management and incident nursing home admissions in older patients with fragility fractures other than the hip—a retrospective cohort study using insurance claims data from Germany
Source: BMC Med. 2025 Apr 29;23:248. doi: 10.1186/s12916-025-04073-3 (PMC12042564; doi:10.1186/s12916-025-04073-3)
Supplement: Supplementary file 1 — Additional file 1: Table 1. The proportion of individuals in the OGCM group for whom the OPS 8 - 550 was claimed separated by fracture type. [file 12916_2025_4073_MOESM1_ESM.docx]

**Additional File 1**: Table 1 The proportion of individuals in the OGCM group for whom the OPS8-550 was claimed separated by fracture type

|  | **Humerus fracture** | **Forearm fracture** | **Pelvis  fracture** | **Vertebral  fracture** |
| --- | --- | --- | --- | --- |
| Men | 17.8% | 7.1% | 25.9% | 18.7% |
| Women | 20.4% | 7.8% | 30.4% | 23.2% |
|  |  |  |  |  |
| Age groups |  |  |  |  |
| 80-85 | 17.7% | 5.4% | 28.8% | 19.9% |
| 85-90 | 21.2% | 9.0% | 30.1% | 23.1% |
| 90+ | 23.8% | 13.3% | 30.4% | 26.4% |
|  |  |  |  |  |
| Overall | 20.0% | 7.8% | 29.7% | 22.1% |
|  |  |  |  |  |
