# Supplementary material for: Orthogeriatric co-management and incident nursing home admissions in older patients with fragility fractures other than the hip—a retrospective cohort study using insurance claims data from Germany
Source: BMC Med. 2025 Apr 29;23:248. doi: 10.1186/s12916-025-04073-3 (PMC12042564; doi:10.1186/s12916-025-04073-3)
Supplement: Supplementary file 2 — Additional file 2: Table 2. Crude incidence rate ratiosfor OGCM versus no OGCM overall and stratified according to fracture site. [file 12916_2025_4073_MOESM2_ESM.docx]

**Additional File 2:** Table 2 Crude incidence rate ratios (IRR) for OGCM versus no OGCM overall and stratified according to fracture site

|  | Humerus fracture | Forearm fracture | Pelvis fracture | Vertebral fracture |
| --- | --- | --- | --- | --- |
|  | IRR (95%CI) | IRR (95%CI) | IRR (95%CI) | IRR (95%CI) |
| Overall | 0.97 (0.92-1.03) | 1.09 (1.01-1.18) | 1.06 (0.99-1.13) | 0.98 (0.93-1.03) |
| *Age groups* | | | | |
| 80-84 | 0.99 (0.89-1.09) | 0.99 (0.85-1.14) | 0.98 (0.87-1.10) | 0.98 (0.89-1.07) |
| 85-89 | 0.98 (0.90-1.08) | 1.11 (0.98-1.26) | 0.98 (0.89-1.09) | 0.97 (0.89-1.05) |
| 90+ | 0.93 (0.83-1.03) | 1.14 (0.99-1.31) | 1.24 (1.11-1.39) | 0.96 (0.86-1.06) |
| *Sex* | | | | |
| Men | 0.97 (0.83-1.14) | 1.10 (0.80-1.51) | 1.25 (1.04-1.50) | 1.01 (0.89-1.13) |
| Women | 0.97 (0.91-1.03) | 1.09 (1.01-1.19) | 1.03 (0.97-1.11) | 0.97 (0.91-1.03) |

No OGCM = reference group; stratification by age and sex
IRR = incidence rate ratio; CI = confidence interval
