# Supplementary material for: Orthogeriatric co-management and incident nursing home admissions in older patients with fragility fractures other than the hip—a retrospective cohort study using insurance claims data from Germany
Source: BMC Med. 2025 Apr 29;23:248. doi: 10.1186/s12916-025-04073-3 (PMC12042564; doi:10.1186/s12916-025-04073-3)
Supplement: Supplementary file 3 — Additional file 3: Fig. S1 A–D. Sensitivity analyses with including surgically treated patients only. [file 12916_2025_4073_MOESM3_ESM.docx]

**Additional File 3**: Figure S1 A-D Sensitivity analyses with including surgically treated patients only

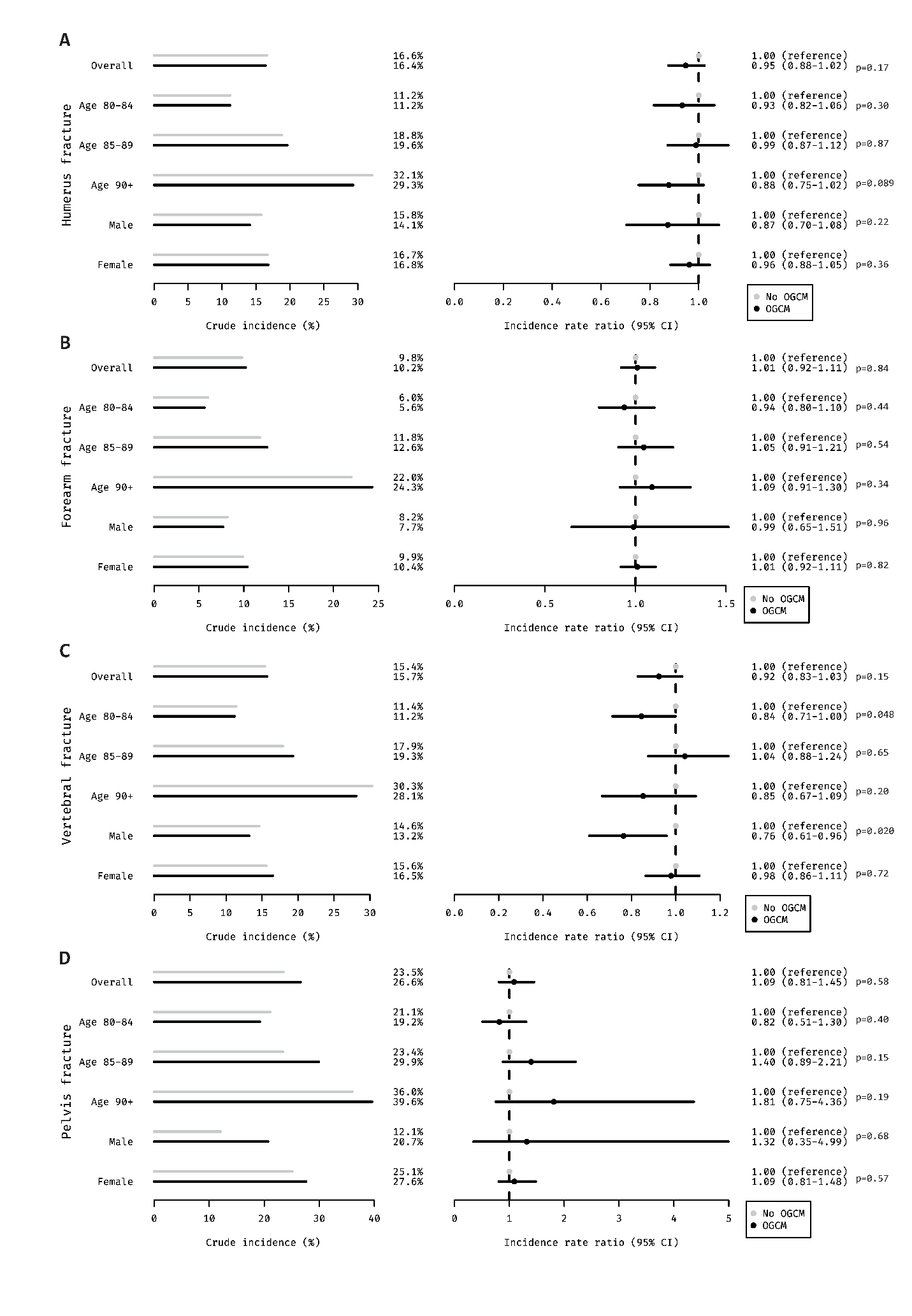


Crude incidences and adjusted rate ratios for OGCM (black) versus no OGCM (grey) overall and stratified by age and sex according to fracture site; A) humerus fracture, B) forearm fracture, C) vertebral fractures, D) pelvic fractures.
Incidence rate ratios are adjusted for age at index fracture, sex, care need in the month prior to fracture, percentage of subacute rehabilitation, size of the index hospital, and Huber comorbidity score.

*OGCM = orthogeriatric co-management; CI = confidence interval*
