# Supplementary material for: Orthogeriatric co-management and incident nursing home admissions in older patients with fragility fractures other than the hip—a retrospective cohort study using insurance claims data from Germany
Source: BMC Med. 2025 Apr 29;23:248. doi: 10.1186/s12916-025-04073-3 (PMC12042564; doi:10.1186/s12916-025-04073-3)
Supplement: Supplementary file 4 — Additional file 4. STROBE checklist. [file 12916_2025_4073_MOESM4_ESM.docx]

STROBE Statement—Checklist of items that should be included in reports of ***cohort studies***

|  | Item No | Recommendation | Page No. | Relevant section in manuscript |
| --- | --- | --- | --- | --- |
| Page **Title and abstract** | 1 | (*a*) Indicate the study’s design with a commonly used term in the title or the abstract | 1 | Title |
|  |  | (*b*) Provide in the abstract an informative and balanced summary of what was done and what was found | 2-3 | Abstract |
| Introduction | | |  |  |
| Background/rationale | 2 | Explain the scientific background and rationale for the investigation being reported | 4-5 | Section ‘background’ |
| Objectives | 3 | State specific objectives, including any prespecified hypotheses | 6 | Final paragraph of section ‘background’ |
| Methods | | |  |  |
| Study design | 4 | Present key elements of study design early in the paper | 6 | First paragraph sub-section ‘study design’ |
| Setting | 5 | Describe the setting, locations, and relevant dates, including periods of recruitment, exposure, follow-up, and data collection | 6-8 | Sub-sections ‘study population’, ‘study exposure’ |
| Participants | 6 | (*a*) Give the eligibility criteria, and the sources and methods of selection of participants. Describe methods of follow-up | 6-7 | Sub-section ‘study population’ |
|  |  | (*b*) For matched studies, give matching criteria and number of exposed and unexposed |  | N/A |
| Variables | 7 | Clearly define all outcomes, exposures, predictors, potential confounders, and effect modifiers. Give diagnostic criteria, if applicable | 8-9 | Sub-sections ‘outcome measures’, ‘covariates’ |
| Data sources/ measurement | 8* | For each variable of interest, give sources of data and details of methods of assessment (measurement). Describe comparability of assessment methods if there is more than one group | 6-7, 8 | Sub-sections ‘study design’, ‘study population’, ‘study exposure’, ‘outcome measures’ |
| Bias | 9 | Describe any efforts to address potential sources of bias |  | Sub-sections ‘study population’, ‘study exposure’; Sub-section ‘strength and limitations’ |
| Study size | 10 | Explain how the study size was arrived at |  | N/A (claims data) |
| Quantitative variables | 11 | Explain how quantitative variables were handled in the analyses. If applicable, describe which groupings were chosen and why |  | Sub-section ‘statistical analysis’ |
| Statistical methods | 12 | (*a*) Describe all statistical methods, including those used to control for confounding | 9 | Sub-section ‘statistical analysis’ |
|  |  | (*b*) Describe any methods used to examine subgroups and interactions | 9 | Sub-section ‘statistical analysis’ |
|  |  | (*c*) Explain how missing data were addressed |  | N/A (claims data) |
|  |  | (*d*) If applicable, explain how loss to follow-up was addressed |  | N/A (claims data) |
|  |  | (*e*) Describe any sensitivity analyses | 9 | Sub-section ‘statistical analysis’ |
| Results | | |  |  |
| Participants | 13* | (a) Report numbers of individuals at each stage of study—eg numbers potentially eligible, examined for eligibility, confirmed eligible, included in the study, completing follow-up, and analysed | 9-11 | First paragraph section ‘results’, Table 1 |
|  |  | (b) Give reasons for non-participation at each stage | No pagination yet | Flow diagram (Fig 1) |
|  |  | (c) Consider use of a flow diagram | No pagination yet | Flow diagram (Fig 1) |
| Descriptive data | 14* | (a) Give characteristics of study participants (eg demographic, clinical, social) and information on exposures and potential confounders | 10-11 | Section ‘results’ second paragraph, Table 1 |
|  |  | (b) Indicate number of participants with missing data for each variable of interest | No pagination yet | Flow diagram (Fig 1) |
|  |  | (c) Summarise follow-up time (eg, average and total amount) |  | Sub-section ‘outcome measure’ |
| Outcome data | 15* | Report numbers of outcome events or summary measures over time | No pagination yet | Figure 2 A-D |
| Main results | 16 | (*a*) Give unadjusted estimates and, if applicable, confounder-adjusted estimates and their precision (eg, 95% confidence interval). Make clear which confounders were adjusted for and why they were included | 11 | Second paragraph; Additional File 2: Table 2, Figure 2 A-D |
|  |  | (*b*) Report category boundaries when continuous variables were categorized |  | N/A |
|  |  | (*c*) If relevant, consider translating estimates of relative risk into absolute risk for a meaningful time period |  | Figure 2 A-D |
| Other analyses | 17 | Report other analyses done—eg analyses of subgroups and interactions, and sensitivity analyses | 11-12 | Sensitivity analyses, Additional File 3: Fig S1 A-D |
| Discussion | | |  |  |
| Key results | 18 | Summarise key results with reference to study objectives | 12 | Section ‘discussion’ first paragraph |
| Limitations | 19 | Discuss limitations of the study, taking into account sources of potential bias or imprecision. Discuss both direction and magnitude of any potential bias | 16-17 | Sub-section ‘strength and limitations’ |
| Interpretation | 20 | Give a cautious overall interpretation of results considering objectives, limitations, multiplicity of analyses, results from similar studies, and other relevant evidence | 13-15, 18 | Section ‘discussion’, sub-section ‘conclusions’ |
| Generalisability | 21 | Discuss the generalisability (external validity) of the study results | 16 | Sub-section ‘strength and limitations’ |
| Other information | | |  |  |
| Funding | 22 | Give the source of funding and the role of the funders for the present study and, if applicable, for the original study on which the present article is based | 20 | Declarations, sub-section ‘funding’ |

* information is given separately for exposed and unexposed groups.
